# European *versus* Asian differences for the associations between paraoxonase‐1 genetic polymorphisms and susceptibility to type 2 diabetes mellitus

**DOI:** 10.1111/jcmm.13453

**Published:** 2018-01-04

**Authors:** Jian‐Quan Luo, Huan Ren, Mou‐Ze Liu, Ping‐Fei Fang, Da‐Xiong Xiang

**Affiliations:** ^1^ Department of Pharmacy The Second Xiangya Hospital Central South University Changsha Hunan China; ^2^ Institute of Clinical Pharmacy Central South University Changsha Hunan China; ^3^ Department of Clinical Pharmacology Xiangya Hospital Central South University Changsha China; ^4^ Hunan Key Laboratory of Pharmacogenetics Institute of Clinical Pharmacology Central South University Changsha China

**Keywords:** type 2 diabetes mellitus, susceptibility, paraoxonase‐1, polymorphism, ethnic difference

## Abstract

Many studies have examined the associations between paraoxonase‐1 (PON1) genetic polymorphisms (Q192R, rs662 and L55M, rs854560) and the susceptibility to type 2 diabetes mellitus (T2DM) across different ethnic populations. However, the evidence for the associations remains inconclusive. In this study, we performed a meta‐analysis to clarify the association of the two *PON1* variants with T2DM risk. We carried out a systematic search of PubMed, Embase, CNKI and Wanfang databases for studies published before June 2017. The pooled odds ratios (ORs) for the association and their corresponding 95% confidence intervals (CIs) were calculated by a random‐ or fixed‐effect model. A total of 50 eligible studies, including 34 and 16 studies were identified for the *PON1* Q192R (rs662) and L55M (rs854560) polymorphism, respectively. As for the *PON1* Q192R polymorphism, the 192R allele was a susceptible factor of T2DM in the South or East Asian population (OR > 1, *P* < 0.05) but represented a protective factor of T2DM in European population (OR = 0.66, 95% CI = 0.45–0.98) under a heterozygous genetic model. With regard to the *PON1* L55M polymorphism, significant protective effects of the 55M allele on T2DM under the heterozygous (OR = 0.77, 95% CI = 0.61–0.97) and dominant (OR = 0.80, 95% CI = 0.65–0.99) genetic models were found in the European population, while no significant associations in the Asian populations under all genetic models (*P* > 0.05). In summary, by a comprehensive meta‐analysis, our results firmly indicated that distinct effects of *PON1* genetic polymorphisms existed in the risk of T2DM across different ethnic backgrounds.

## Introduction

The rise of diabetes prevalence poses one of the important challenges to global health. It is estimated that approximately 422 million adults were diagnosed with the disease in 2014 worldwide 1. Diabetes is one of the main causes of cardiovascular disease, blindness and kidney failure and is the sixth leading driver of disability 2. Therefore, the prevention and control of diabetes are growing up to be an ever‐increasing global health priority 3. Type 2 diabetes mellitus (T2DM) comprises the majority of cases of diabetes around the world. T2DM is a metabolic disorder of multifactorial aetiology involving many environmental factors and genetic variants 4, 5.

Human paraoxonase‐1 (PON1) is a calcium‐dependent 45‐kD glycoprotein composed of 355 amino acids. The esterase is synthesized mainly by the liver and secreted into the circulation where it associates with high‐density lipoprotein (HDL) and assists in the antioxidant effect of preventing oxidation of low‐density lipoprotein (LDL). PON1 in human beings is encoded by the *PON1* gene which maps to the long arm of chromosome 7 (q21‐22). It has been observed that serum PON1 activity has an important role in susceptibility and progression of T2DM 6, 7.

Single nucleotide polymorphisms (SNPs) in the *PON1* gene can significantly account for the catalytic ability of the enzyme. A missense SNP at position 192 (glycine (Q) to arginine (R) substitution) (rs662) is an important determinant of the PON1 activity 8. Although the R‐alloenzyme is more active towards some substrates, for example paraoxon, other substrates such as diazoxon and sarin are hydrolysed more rapidly by the Q‐alloenzyme 9. In addition, the *PON1* Q192R polymorphism was the major determinant of individual variation in the ability of HDL in protecting LDL against lipid peroxidation. For example, the Q‐alloenzyme confers least ability 10. Another SNP in the coding region causes a leucine (L) to methionine (M) substitution at position 55 (rs854560), which may also affect the PON1 activity and levels 11.

As Ikeda *et al*. first found that serum PON activity was significantly decreased in the patients with T2DM 12, a large number of studies have been conducted over the last two decades to investigate the association of Q192R (rs662) and L55M (rs854560) polymorphism in *PON1* gene with susceptibility to T2DM. However, the previously published results remain controversial. Hence, to firmly elucidate the association between *PON1* genetic polymorphisms (Q192R, rs662 and L55M, rs854560) and the risk of T2DM, we conducted a systematic review and meta‐analysis of data from 50 studies and also established the association according to the ethnicity.

## Materials and methods

### Search strategy and inclusion criteria

A systematic search was conducted in the electronic databases PubMed, Embase, China National Knowledge Infrastructure (CNKI) and Wanfang Data, and all relevant articles were published in English or Chinese from their starting dates to June 2017. The search strategy used the following keywords relating to the paraoxonase‐1 gene (‘paraoxonase‐1′, ‘PON1′) or variations (*e.g*. ‘mutation’, ‘polymorphism’, ‘single nucleotide polymorphism’, ‘SNP’, ‘variant’, ‘variation’) in combination with TD2M (*e.g*. ‘Diabetes Mellitus, Type 2′, ‘Noninsulin‐Dependent Diabetes Mellitus’, ‘Type 2 Diabetes’, ‘Diabetes Mellitus, Noninsulin‐Dependent’). We supplemented this search by reviewing the cited references for all possible studies.

All identified abstracts were carefully reviewed by two investigators (J. Q. Luo, H. Ren) independently for eligibility. The inclusion criteria were as follows: (*i*) case‐control design, regardless of sample size; (*ii*) study assessing the associations between Q192R (rs662) and L55M (rs854560) of *PON1* gene and type 2 diabetes; (*iii*) numbers for the *PON1* genotypes could be available or calculated in case and control groups; and (*iv*) genotype distribution in the controls was in Hardy‐Weinberg equilibrium (HWE). If the two investigators (J. Q. Luo, H. Ren) disagreed about the eligibility of an article, it was resolved by consensus with a third reviewer (M. Z. Liu).

### Data extraction

For the eligible articles included in this study, data were also extracted by two reviewers (J. Q. Luo, H. Ren), who reached a consensus on all of the data extraction items. The following information was extracted from each study: name of the first author, publication year, country of the study, ethnicity of the population, genotype and allele distributions in case and control groups, and also sample size, mean age and gender distribution in case and control groups.

### Statistical analysis

The goodness‐of‐fit chi‐square analysis was used to test the HWE of the genotype distribution of controls. The distribution was considered deviated significantly from HWE with *P* < 0.05. The pooled odds ratio (OR) with 95% confidence interval (CI) was used to evaluate the strength of association in the allelic, homozygous, heterozygous, recessive and dominant models, respectively. The statistical significance of the pooled estimates of the OR was determined by the *Z* test. The Cochran's *Q* test and *I* ^2^ metric were performed to examine the possibility of between‐study heterogeneity. Heterogeneity was considered to be statistically significant at *P* < 0.05 for the Q statistic and *I* ^2^ > 50% for the *I* ^2^ metric 13. If substantial heterogeneity existed, random effect model (the DerSimonian and Laird method) was selected as the pooling method. Otherwise, the fixed‐effect model (the Mantel‐Haenszel method) was adopted. Subgroup analysis based on ethnicity (categorized as Europeans, East Asians, South Asians and Canadian Aboriginal) and meta‐regression with restricted maximum likelihood estimation were conducted to assess the sources of heterogeneity across the studies. Potential publication bias was assessed by Begg's test and Egger's test 14, 15, with *P* < 0.05 considered representative of significant publication bias. All statistical analyses were performed with STATA version 12.0 (Stata, College Station, TX, USA).

## Results

### Description of eligible studies

**Figure 1:**
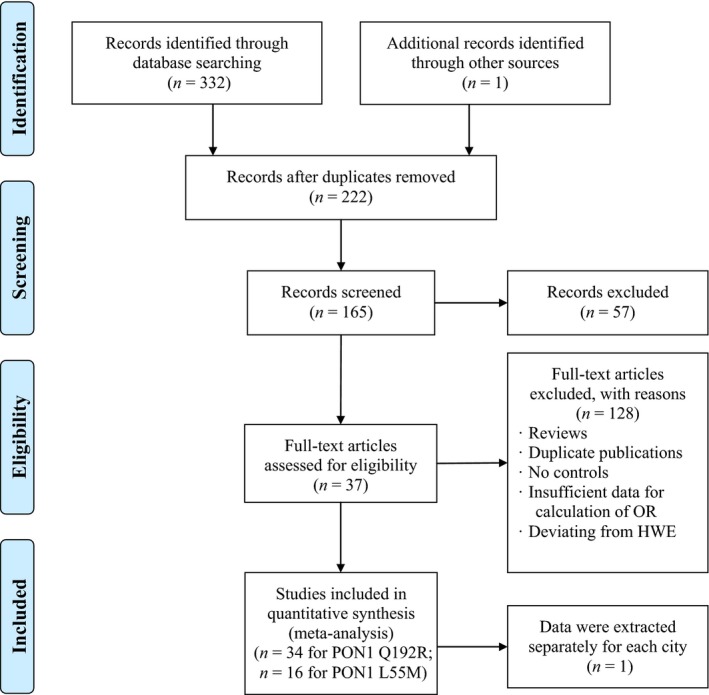
Flow diagram of the search strategy and study selection. The terms ‘*n*’ in the boxes represent the number of corresponding studies.

The initial screening yielded 332 articles, and 1 article was found to be eligible by reviewing the cited references. A total of 111 articles were excluded because of duplicate publication. Then, 57 articles were excluded from screening based on the titles and/or abstracts. Finally, 37 articles 16, 17, 18, 19, 20, 21, 22, 23, 24, 25, 26, 27, 28, 29, 30, 31, 32, 33, 34, 35, 36, 37, 38, 39, 40, 41, 42, 43, 44, 45, 46, 47, 48, 49, 50, 51, 52 involving 50 eligible studies were included in the current meta‐analysis according to the study inclusion criteria (Fig. 1). All the included articles were case‐control designs with sample sizes varied from 61 to 593. A total of 34 and 16 eligible studies were identified for the *PON1* Q192R (rs662) and L55M (rs854560) polymorphism, respectively. The general characteristics of the studies included in the meta‐analysis are presented in Table 1.

**Table 1:** Characteristics of the included studies of the association of the PON1 Q192R and L55M genetic polymorphism with type 2 diabetes

Study per SNP	Year	Country (Population)^a^	Male/Female		Age(years)		Sample size^b^	Case genotypes or alleles^c^					Control genotypes or alleles^c^					MAF^d^	HWE P^e^
			Case	Control	Case	Control		11	12	22	1	2	11	12	22	1	2		
PON1 Q 192R																			
Mackness	1998	United Kingdom(EUR)	162/90	147/135	59.1 ± 11.3	42.2 ± 12.2	252/282	117	99	34	333	167	156	99	24	411	147	0.263	0.153
Sakai	1998	Japan(EAS)	65/74	179/61	61.7 ± 13.6	48.0 ± 8.7	139/240	14	63	62	91	187	24	102	114	150	330	0.688	0.866
Fanella	2000	Canada	NA	NA	44.5 ± 15.4	25.4 ± 12.6	115/478	74	36	5	184	46	276	175	27	727	229	0.240	0.915
Koch(a)	2001	Germany(EUR)	NA	NA	NA	NA	39/202	25	12	2	62	16	102	87	13	291	113	0.280	0.328
Koch(b)	2001	Germany(EUR)	NA	NA	NA	NA	36/113	17	19	0	53	19	62	45	6	169	57	0.252	0.554
Sampson	2001	United Kingdom(EUR)	18/22	14/16	56.6 ± 7.3	52.0 ± 7.4	40/30	17	16	2	50	20	12	11	3	35	17	0.327	0.844
Letellier	2002	France(EUR)	57/39	52/53	56.8 ± 10.7	46.7 ± 10.9	167/105	92	67	8	251	83	40	50	14	130	78	0.375	0.794
Hu YM	2003	China(EAS)	95/57	83/45	58.5 ± 12.1	54 ± 11.5	152/128	30	77	45	137	167	25	74	29	124	132	0.516	0.075
Zhang	2003	Japan(EAS)	39/17	61/28	64.5 ± 7.5	62.7 ± 8.3	56/89	10	23	23	43	69	7	33	49	47	131	0.736	0.665
Ma RX	2003	China(EAS)	40/40	55/49	63.0 ± 8.0	64.0 ± 7.0	176/104	16	84	76	116	236	22	48	34	92	116	0.558	0.511
Wang Y	2003	China(EAS)	24/12	29/9	64.8 ± 11.9	70.8 ± 10.8	75/38	18	41	16	77	73	15	18	5	48	28	0.368	0.912
Pu X	2003	China(EAS)	14/16	55/45	67.0 ± 5.0	64.0 ± 4.0	100/100	6	64	30	76	124	18	52	30	88	112	0.56	0.581
Hao YL	2003	China(EAS)	50/55	44/36	54.5 ± 10.2	51.6 ± 6.3	187/80	26	86	75	138	236	6	32	42	44	116	0.725	0.978
Ren T	2003	China(EAS)	65/55	57/26	NA	NA	112/83	22	95	78	139	251	9	42	29	60	100	0.625	0.283
Li SY	2004	China(EAS)	22/14	21/12	56.0 ± 8.0	57.0 ± 11.0	63/33	16	24	23	56	70	13	13	7	39	27	0.409	0.287
Zhang Z	2004	China(EAS)	30/26	49/31	63.6 ± 11.4	63.7 ± 11.5	116/80	16	41	59	73	159	12	41	27	65	95	0.594	0.577
Deng YG	2004	China(EAS)	37/43	45/45	60.1 ± 2.7	54.8 ± 3.7	169/90	20	57	90	97	237	13	44	33	70	110	0.611	0.786
Sun YD	2005	China(EAS)	92/85	50/47	64.5 ± 10.3	62.4 ± 10.9	162/97	53	161	95	267	351	14	56	27	84	110	0.567	0.083
Mastorikou	2006	United Kingdom(EUR)	21/15	10/9	57.7 ± 5.2	57.7 ± 4.8	36/19	NA	NA	NA	48	24	NA	NA	NA	28	10	0.263	NA
Qi L	2007	China(EAS)	44/49	35/54	56.6 ± 7.0	57.9 ± 6.8	183/89	32	97	54	161	205	18	42	29	78	100	0.562	0.695
Shi GH	2007	China(EAS)	49/43	43/38	60.9 ± 7.3	62.4 ± 6.3	179/81	33	69	77	135	223	12	38	31	62	100	0.617	0.949
Irace	2008	Italy(EUR)	NA	NA	55.2 ± 9.2	55.9 ± 6.6	118/65	64	42	12	170	66	26	31	8	83	47	0.362	0.790
Unür	2008	Turkey(EUR)	20/31	27/26	52.5 ± 5.6	55.5 ± 8.0	51/53	31	14	6	76	26	25	22	6	72	34	0.321	0.730
Flekac	2008	Czech(EUR)	114/132	55/45	58 ± 18	41 ± 9	246/110	177	64	5	418	74	32	54	24	118	102	0.464	0.892
Gorshunska	2009	Ukraine(EUR)	28/68	86/47	56.2 ± 1.4	55.3 ± 2.5	96/123	55	25	16	135	57	48	64	11	160	86	0.350	0.110
Ergun	2011	Turkey(EUR)	NA	NA	59 ± 9.63	47 ± 6.53	171/80	91	50	30	232	110	38	31	11	107	53	0.331	0.262
Bhaskar	2011	India(SAS)	NA	NA	NA	NA	310/120	71	184	55	326	294	40	66	14	146	94	0.392	0.091
Gupta	2011	India(SAS)	126/124	151/149	47.4 ± 11.3	43.1 ± 10.7	250/300	81	126	43	288	212	168	108	24	444	156	0.26	0.264
Chen XJ	2011	China(EAS)	50/47	55/50	59.9 ± 10.6	58.7 ± 5.7	210/105	23	109	78	155	265	12	58	35	82	128	0.610	0.100
Elnoamany	2012	Egypt(EUR)	33/12	30/10	51.11 ± 6.7	50.19 ± 5.5	93/40	42	28	23	112	74	25	13	2	63	17	0.213	0.855
Zheng YQ	2012	China(EAS)	51/39	70/66	57.1 ± 12.0	45.5 ± 13.3	184/136	21	66	97	108	260	19	57	60	95	177	0.651	0.363
Gokcen	2013	Turkey(EUR)	20/30	16/14	60.8 ± 9.4	54.2 ± 8.1	50/30	18	25	7	61	39	8	14	8	30	30	0.500	0.715
Shao ZY	2014	China(EAS)	94/83	111/95	63.3 ± 10.9	61.3 ± 11.0	379/206	50	173	156	273	485	35	94	77	164	248	0.602	0.493
Du WL	2015	China(EAS)	28/33	28/36	54.9 ± 12.7	37.7 ± 14.5	125/64	7	43	75	57	193	5	22	37	32	96	0.75	0.505
PON1 L55M																			
Ikeda	1998	Japan(EAS)	53/55	82/79	58 ± 7	57 ± 8	108/161	95	10	3	200	16	142	19	0	303	19	0.059	0.426
Malin	1999	Finnish(EUR)	NA	NA	NA	NA	93/106	33	49	11	115	71	38	54	14	130	82	0.387	0.447
Fanella	2000	Canada	NA	NA	44.5 ± 15.4	25.4 ± 12.6	115/478	113	2	0	228	2	471	7	0	949	7	0.007	0.872
Letellier	2002	France(EUR)	57/39	52/53	56.8 ± 10.7	46.7 ± 10.9	167/105	65	81	20	211	121	40	52	12	132	76	0.366	0.426
Ren T	2003	China(EAS)	65/55	57/26	NA	NA	184/83	177	7	0	361	7	70	2	0	142	2	0.013	0.905
Agachan	2004	Turkish(EUR)	122/91	57/59	59.9 ± 11.6	58.6 ± 16.0	213/116	111	86	8	308	102	51	51	7	153	65	0.298	0.218
Sampson	2005	United Kingdom(EUR)	38/20	18/32	NA	NA	58/50	26	32	32	NA	NA	21	28	28	NA	NA	NA	NA
Mastorikou	2006	United Kingdom(EUR)	21/15	10/9	57.7 ± 5.2	57.7 ± 4.8	36/19	NA	NA	NA	49	23	NA	NA	NA	24	14	0.368	NA
Sun YD	2006	China(EAS)	92/85	50/47	64.5 ± 10.3	62.4 ± 10.9	294/91	121	150	23	392	196	38	45	8	121	61	0.335	0.296
Shao HQ	2006	China(EAS)	29/21	60/60	61.5 ± 3.3	55.0 ± 2.3	92/120	85	7	0	177	7	109	11	0	229	11	0.046	0.599
Flekac	2008	Czech(EUR)	114/132	55/45	58 ± 18	41 ± 9	246/110	84	118	44	286	206	30	55	25	115	105	0.477	0.983
Unür	2008	Turkey(EUR)	20/31	27/26	52.5 ± 5.6	55.5 ± 8.0	51/53	43	5	3	91	11	28	20	5	76	30	0.283	0.609
Altuner	2011	Turkey(EUR)	56/44	21/29	54.4 ± 2.8	44.04 ± 1.1	100/50	43	44	13	130	70	21	25	4	67	33	0.33	0.355
Ergun	2011	Turkish(EUR)	NA	NA	59 ± 9.6	47 ± 6.5	171/80	35	45	91	115	227	16	30	34	62	98	0.613	0.060
Gupta	2011	India(SAS)	126/124	151/149	47.4 ± 11.3	43.1 ± 10.7	250/300	176	69	5	421	79	193	101	6	487	113	0.188	0.080
Zheng YQ	2012	China(EAS)	51/39	70/66	57.1 ± 12.0	45.5 ± 13.3	184/136	168	15	1	351	17	124	11	1	259	13	0.048	0.194
Shao ZY	2014	China(EAS)	94/83	111/95	63.3 ± 10.9	61.3 ± 11.0	379/206	339	34	6	712	46	184	20	2	388	24	0.058	0.099

a The population codes of EAS, EUR and SAS mean East Asian, European and South Asian, respectively.

b Sample size means the case‐control groups.

c For the PON1 Q192R, 11: QQ, 12: QR, 22: RR; for the PON1 L55M, 11: LL, 12:LM, 22: MM.

d MAF, minor allele frequency; NA, not available.

e HWE, Hardy‐Weinberg equilibrium.

### Quantitative synthesis of the association between *PON1* Q912R polymorphism and T2DM

**Figure 2:**
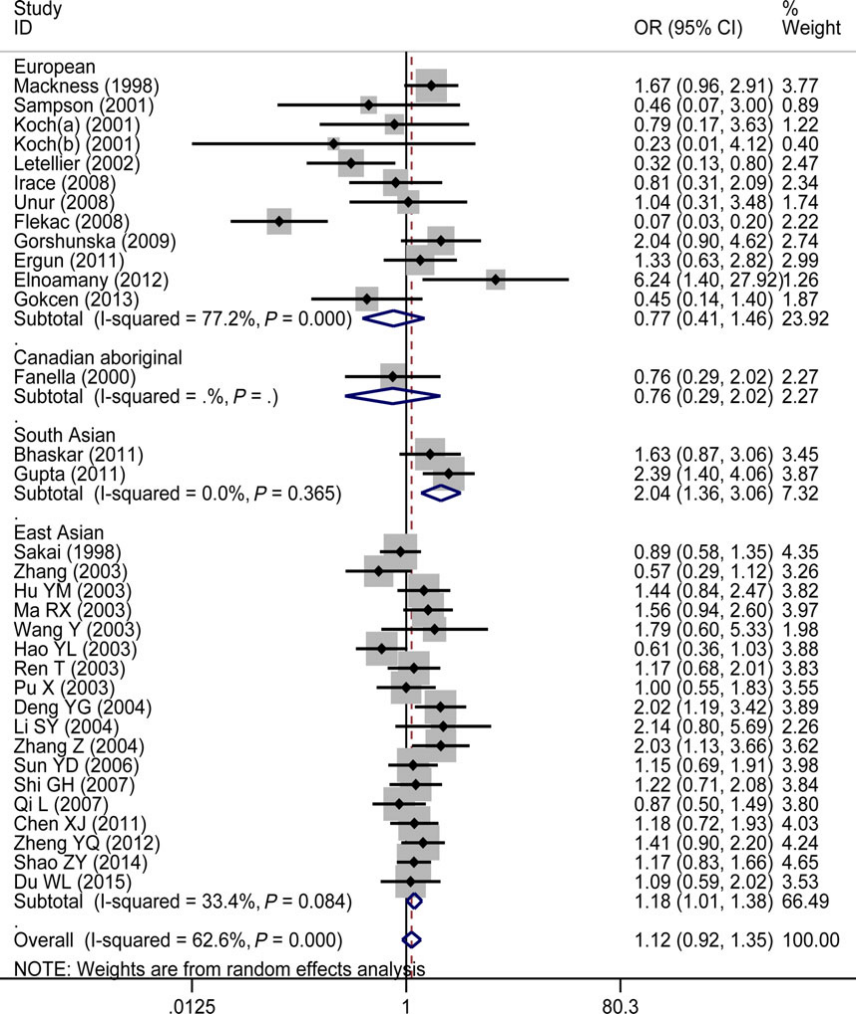
Forest plot for PON1 Q192R polymorphism under a recessive genetic model stratified by ethnicity in studies with type 2 diabetes patients.

The results of the meta‐analysis of *PON1* Q912R polymorphism are summarized in detail in Table 2 and Figure 2. In the overall population, the pooled meta‐analysis revealed that there were no significant associations between the *PON1* Q912R genetic polymorphism and T2DM under all genetic models: allelic (OR = 1.02, 95% CI = 0.87–1.20; *P* = 0.786), homozygous (OR = 1.08, 95% CI = 0.81–1.45; *P* = 0.596), heterozygous (OR = 0.93, 95% CI = 0.75–1.17; *P* = 0.544), recessive (OR = 1.12, 95% CI = 0.92–1.35; *P* = 0.259) and dominant (OR = 0.99, 95% CI = 0.78–1.26; *P* = 0.921).

**Table 2:** Summary of meta‐analysis of the association of the PON1 Q192R and L55M genetic polymorphism with type 2 diabetes

Genetic model^a^	PON1 R192R							PON1 L55M						
	Pooled OR (95%CI)	*Z*	*P* b	*N* c	Model^d^	*I* ^2^%	*P*_hetero_	Pooled OR(95%CI)	*Z*	*P* b	*N* c	Model^d^	*I* ^2^%	*P*_hetero_
Allelic	1.02 (0.87–1.20)	0.27	0.786	34	R	81.8%	0.000	0.91 (0.81–1.02)	1.56	0.118	16	F	2.3%	0.427
EUR subgroup	0.80 (0.56–1.16)	1.16	0.246	13	R	87.1%	0.000	0.89 (0.77–1.03)	1.52	0.129	8	F	45.7%	0.075
Canadian Aboriginal	0.79 (0.56–1.13)	1.27	0.203	1	NA	NA	NA	1.19 (0.25–5.76)	0.22	0.830	1	NA	NA	NA
SAS subgroup	1.73 (1.17–2.56)	2.72	0.007^b^	2	R	74.8%	0.046	0.81 (0.59–1.11)	1.32	0.187	1	NA	NA	NA
EAS subgroup	1.14 (1.01–1.28)	2.1	0.036^b^	18	R	43.4%	0.026	1.03 (0.81–1.31)	0.22	0.825	6	F	0.0%	0.978
Recessive	1.12 (0.92–1.35)	1.13	0.259	33	R	62.6%	0.000	1.05 (0.81–1.35)	0.35	0.729	12	F	0.0%	0.630
EUR subgroup	0.77 (0.41–1.46)	0.80	0.425	12	R	77.2%	0.000	1.02 (0.76–1.35)	0.11	0.912	7	F	0.0%	0.427
Canadian Aboriginal	0.76 (0.29–2.02)	0.55	0.580	1	NA	NA	NA	NA	NA	NA	NA	NA	NA	NA
SAS subgroup	2.03 (1.35–3.05)	3.39	0.001^b^	2	F	0.0%	0.365	1.00 (0.30–3.32)	0.00	1.000	1	NA	NA	NA
EAS subgroup	1.18 (1.04–1.33)	2.6	0.009^b^	18	F	33.4%	0.084	1.25 (0.63–2.47)	0.63	0.529	4	F	0.0%	0.405
Dominant	0.99 (0.78–1.26)	0.10	0.921	33	R	78.4%	0.000	0.85 (0.73–0.99)	2.15	0.032^b^	16	F	0.0%	0.628
EUR subgroup	0.69 (0.45–1.06)	1.70	0.089	12	R	83.5%	0.000	0.80 (0.65–0.99)	2.10	0.036^b^	8	F	31.9%	0.173
Canadian Aboriginal	0.76 (0.50–1.16)	1.29	0.197	1	NA	NA	NA	1.19 (0.24–5.81)	0.22	0.829	1	NA	NA	NA
SAS subgroup	2.26 (1.72–2.98)	5.78	0.000^b^	2	F	57.9%	0.123	0.76 (0.53–1.09)	1.51	0.132	1	NA	NA	NA
EAS subgroup	1.18 (0.99–1.39)	1.88	0.060	18	F	30.4%	0.108	1.00 (0.75–1.33)	0	0.997	6	F	0.0%	0.997
Homozygous	1.08 (0.81–1.45)	0.53	0.596	33	R	72.1%	0.000	0.92 (0.69–1.23)	0.56	0.577	12	F	0.0%	0.700
EUR subgroup	0.63 (0.30–1.32)	1.22	0.222	12	R	81.6%	0.000	0.85 (0.61–1.19)	0.94	0.348	7	F	0.0%	0.562
Canadian Aboriginal	0.69 (0.26–1.86)	0.73	0.463	1	NA	NA	NA	NA	NA	NA	NA	NA	NA	NA
SAS subgroup	3.01 (1.93–4.67)	4.88	0.000^b^	2	F	21.2%	0.260	0.91 (0.27–3.05)	0.15	0.883	1	NA	NA	NA
EAS subgroup	1.28 (1.06–1.54)	2.54	0.011^b^	18	F	34.5%	0.075	1.28 (0.64–2.59)	0.7	0.487	4	F	0.0%	0.431
Heterozygous	0.93 (0.75–1.17)	0.61	0.544	33	R	71.4%	0.000	0.82 (0.70–0.97)	2.39	0.017^b^	15	F	0.0%	0.609
EUR subgroup	0.66 (0.45–0.98)	2.09	0.037^b^	12	R	75.8%	0.000	0.77 (0.61–0.97)	2.24	0.025^b^	7	F	37.7%	0.141
Canadian Aboriginal	0.77 (0.49–1.19)	1.18	0.239	1	NA	NA	NA	1.19 (0.24–5.81)	0.22	0.829	1	NA	NA	NA
SAS subgroup	2.07 (1.54–2.76)	4.89	0.000^b^	2	F	49.1%	0.161	0.75 (0.52–1.08)	1.54	0.124	1	NA	NA	NA
EAS subgroup	1.09 (0.91–1.30)	0.95	0.341	18	F	18.9%	0.228	0.96 (0.72–1.29)	0.28	0.778	6	F	0.0%	0.984

a The population codes of EAS, EUR and SAS mean East Asian, European and South Asian, respectively.

b *P* < 0.05.

c *N* means the number of eligible studies for the meta‐analysis.

d F, fixed‐effects model; NA, not available; R, random‐effects model.

When we performed subgroup analyses stratified by ethnicity, the distinct effects in different ethnic populations were observed under all genetic models. Significant associations between *PON1* Q912R genetic polymorphism and T2DM presented in the South Asian subgroup (under all genetic models) and East Asian subgroup (under four genetic models), while no significant associations were shown in the Canadian Aboriginal subgroup and in the European subgroup under the allelic, homozygous, recessive and dominant genetic models. By contrast, the significant association for the European subgroup under the heterozygous genetic model showed the 192R allele represented a protective factor of T2DM (OR = 0.66, 95% CI = 0.45–0.98; *P* = 0.037), but a risk factor for T2DM in South Asian subgroup.

### Quantitative synthesis of the association between *PON1* L55M polymorphism and T2DM

**Figure 3:**
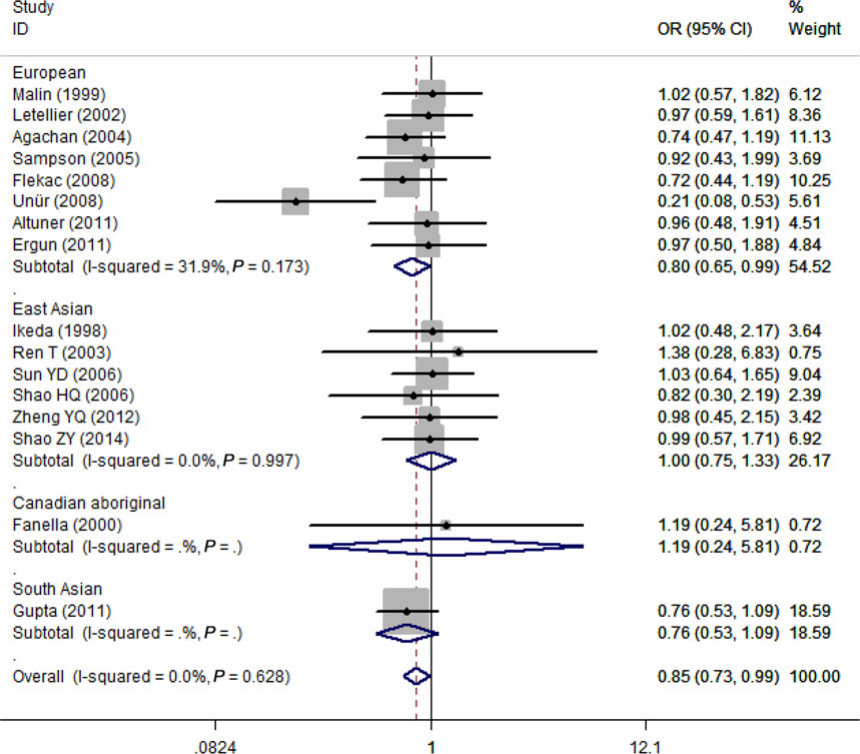
Forest plot for PON1 L55M polymorphism under a dominant genetic model stratified by ethnicity in studies with type 2 diabetes patients.

The results of the meta‐analysis of *PON1* L55M polymorphism are summarized in detail in Table 2 and Figure 3. In the overall population, the associations between the *PON1* L55M genetic polymorphism and T2DM did not reach statistically significant under the allelic genetic model (OR = 0.91, 95% CI = 0.81–1.02; *P* = 0.118), homozygous genetic model (OR = 0.92, 95% CI = 0.69–1.23; *P* = 0.577) and recessive genetic model (OR = 1.05, 95% CI = 0.81–1.35; *P* = 0.729). However, significant associations were found under a heterozygous genetic model (OR = 0.82, 95% CI = 0.70–0.97; *P* = 0.017) and a dominant genetic model (OR = 0.85, 95% CI = 0.73–0.99; *P* = 0.032).

In subgroup analyses based on ethnicity, the distinct effects in different ethnic populations were also presented for the *PON1* L55M genetic polymorphism. There were significant protective effects of L allele on T2DM in the European subgroup under the heterozygous (OR = 0.77, 95% CI = 0.61–0.97; *P* = 0.025) and dominant (OR = 0.80, 95% CI = 0.65–0.99; *P* = 0.036) genetic models, while no significant results were found in the South Asian, East Asian and Canadian Aboriginal subgroup under all genetic models.

### Sources of heterogeneity

There was significant heterogeneity in the overall meta‐analysis of *PON1* Q912R polymorphism under all of the genetic models (*P*_heterogeneity_ < 0.05, *I* ^2^ > 50%). Subgroup analysis stratified by ethnicity indicated that heterogeneity was significantly reduced in the South Asian and East Asian subgroup, while was increased in the European subgroup. Therefore, ethnicity may be one of the sources of heterogeneity between studies for the *PON1* Q912R polymorphism in the overall meta‐analysis.

Because substantial heterogeneity still existed in the European subgroup under all genetic models, meta‐regression was used to explore the source of this heterogeneity. The following three covariates were taken into consideration: publication year, MAF (minor allele frequency) in controls and sample size in the subsequent meta‐regression (Table 3). The results of meta‐regression analysis showed that MAF in the control group could explain the observed between‐study heterogeneity. The proportion of between‐study variance explained by the MAF covariate ranges from 67.81 to 93.06%, depending on the genetic models. However, no significant effects were accounted for by the covariates sample size and publication year under all genetic models.

**Table 3:** The meta‐regression results among the European population under all genetic model for the PON1 Q192R genetic polymorphism

Genetic model	Covariates	Coefficient	Standard Error	*T*‐value	*P*‐value	95% confidence interval	Adjusted *R*‐squared
Heterozygous	MAF in controls	−6.03742	1.641809	−3.68	0.004^a^	−9.6956∼−2.37924	81.00%
	Sample size	−0.00028	0.001466	−0.19	0.854	−0.00354∼0.002991	−14.64%
	Publication year	−0.04524	0.03562	−1.27	0.233	−0.12461∼0.034129	13.01%
Allelic	MAF in controls	−6.41229	1.281261	−5	0.000^a^	−9.23233∼−3.59225	80.80%
	Sample size	−0.00045	0.001437	−0.32	0.759	−0.00366∼0.002749	−11.10%
	Publication year	−0.00646	0.037309	−0.17	0.866	−0.08858∼0.075655	−9.84%
Homozygous	MAF in controls	−12.2995	3.278749	−3.75	0.004^a^	−19.605∼−4.99395	73.94%
	Sample size	−0.00058	0.003051	−0.19	0.853	−0.00738∼0.006216	−13.55%
	Publication year	0.023242	0.081639	0.28	0.782	−0.15866∼0.205145	−13.07%
Dominant	MAF in controls	−7.93603	1.488279	−5.33	0.000^a^	−11.2521∼−4.61994	93.06%
	Sample size	−0.00049	0.001638	−0.3	0.771	−0.00414∼0.003161	−12.29%
	Publication year	−0.02767	0.042206	−0.66	0.527	−0.12171∼0.066367	−4.49%
Recessive	MAF in controls	−10.0113	3.071858	−3.26	0.009^a^	−16.8558∼−3.16679	67.81%
	Sample size	−0.00047	0.002671	−0.18	0.864	−0.00642∼0.005483	−14.87%
	Publication year	0.035213	0.071726	0.49	0.634	−0.1246∼0.19503	−13.82%

a *P* < 0.05.

MAF, minor allele frequency; Coefficient: regression coefficient. The regression coefficients were the estimated increase in the lnOR per unit increase in the covariates.

In contrast, no significant heterogeneity in the overall meta‐analysis of *PON1* L55M polymorphism was showed under all genetic models (*P*_heterogeneity_ > 0.1, *I* ^2^ = 0%). Subgroup analysis stratified by ethnicity also indicated that no substantial between‐study heterogeneity was found in the Asian subgroup (*P*_heterogeneity_ > 0.1, *I* ^2^ = 0%) and in the European subgroup (*P*_heterogeneity_ > 0.05, *I* ^2^ < 50%) under all genetic models.

### Publication bias evaluation

**Figure 4:**
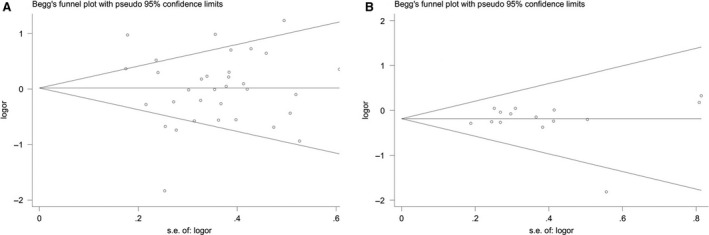
Begg's funnel plot for studies of the association between type 2 diabetes and PON1 Q192R polymorphism under a dominant genetic model (**A**) and PON1 L55M polymorphism under a heterozygous genetic model (**B**).

Publication bias of the individual articles was evaluated by using the Begg's funnel plot (Fig. 4) and Egger's test. For the *PON1* Q192R meta‐analysis (Fig. 4A), no obvious publication bias was visualized in the shape of the funnel plot under all genetic models. Additionally, no evidence of significant publication bias was detected by the Egger's test (*P* = 0.257 for allelic genetic model; *P* = 0.452 for heterozygous genetic model; *P* = 0.527 for dominant genetic model; and *P* = 0.197 for recessive genetic model). However, there was marginal significant publication bias for the homozygous genetic model (*P* = 0.047).

For the *PON1* L55R meta‐analysis (Fig. 4B), there is also no obvious publication bias in the shape of the funnel plot under all genetic models. No evidence of significant publication bias was also detected by the Egger's test (*P* = 0.961 for allelic genetic model; *P* = 0.719 for heterozygous genetic model; *P* = 0.309 for homozygous genetic model; *P* = 0.871 for dominant genetic model; and *P* = 0.628 for recessive genetic model) yet.

## Discussion

So far, the associations between *PON1* genetic polymorphisms and T2DM were conflicting in the previous studies. This is partly because some previous case‐control studies have been too small to be reliable. Thus, our meta‐analysis could overcome the limitations of single study by pooling the individual dataset and provide more reliable results.

In the overall meta‐analysis of the *PON1* Q192R polymorphism, no significant association, but strong between‐study heterogeneity, was observed. To address the substantial heterogeneity, we divided the total samples into four subgroups, that is white European, Canadian Aboriginal, South and East Asians. Stratified analyses by ethnicity yielded a significant association of the *PON1* Q192R polymorphism with T2DM in South Asian and East Asian populations and, conversely, no association of the *PON1* Q192R polymorphism with T2DM in European populations under the allelic, homozygous, recessive and dominant genetic models. In addition, the 192R allele was a susceptible factor of T2DM in the Asian population but represented a protective factor of T2DM in European population under a heterozygous genetic model.

In the overall meta‐analysis of the *PON1* L55M polymorphism, no significance between‐study heterogeneity was observed. The distinct effects across different ethnic backgrounds also presented in the subgroup analysis based on ethnicity. For example, significant protective effects of the 55M allele on T2DM under the heterozygous and dominant genetic models were found in the European population, while no significant results in the Asian populations under all genetic models. Interestingly, the associations of the two *PON1* SNPs in our study were generally very similar in South and East Asians, although Asia is known to harbour genetically different origins 53. In Canadian population, only one study investigated the association between the two *PON1* SNPs and risk of T2DM, and no significant associations were found in all genetic models. Therefore, it was inferred that the 192R or 55M allele may decrease the risk of developing T2DM in European ancestry population, whereas the 192R increase the risk of T2DM in the South Asian and East Asian populations.

To our knowledge, this is the largest study to underline the importance of ethnicity in the association between *PON1* genetic variations and T2DM by a comprehensive meta‐analysis. The question remaining to be addressed is how the *PON1* Q192R and L55M variants can exert an impact on T2DM with ethnic difference. One potential explanation is that different populations might have experienced very diverse lifestyle and environmental factors during their long‐period evolution. The PON1 activity may be influenced by several environmental and lifestyle impacts, such as cigarette smoking 54, alcohol intake 55 and physical activity 56. Another possible explanation may be the ethical differences in the distribution of the *PON1* Q192R and L55M (rs854560) polymorphisms. Nevertheless, the precise mechanism deserves to be investigated in the future.

**Figure 5:**
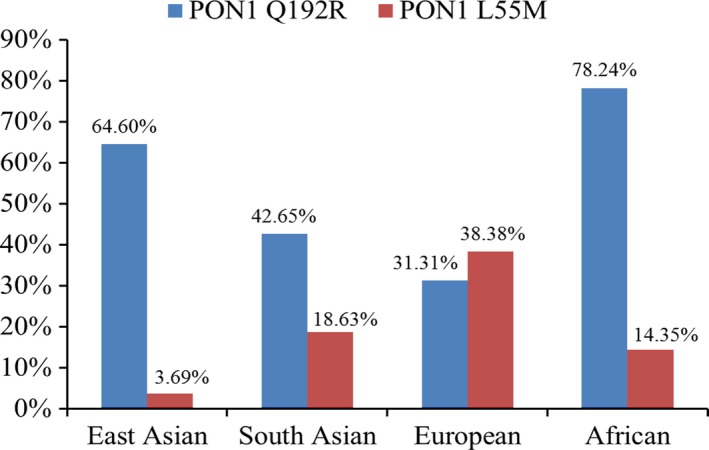
The frequency of PON1 Q192R and L55M among the different ethnicities. The data were summarized according to the 1000 genomes database. East Asian referred to the Chinese and Japanese; South Asian was from India; European referred to Utah Residents (CEPH) with Northern and Western European Ancestry; African was from Yoruba in Ibadan, Nigeria.

According to the included studies among different countries 16, 17, 18, 19, 20, 21, 22, 23, 24, 25, 26, 27, 28, 29, 30, 31, 32, 33, 34, 35, 36, 37, 38, 39, 40, 41, 42, 43, 44, 45, 46, 47, 48, 49, 50, 51, 52 and the 1000 genomes database (https://www.ncbi.nlm.nih.gov/variation/tools/1000genomes/), there are huge racial and regional differences in the distribution of the *PON1* Q192R (rs662) and L55M (rs854560) genetic polymorphisms (Fig. 5). For the *PON1* Q192R polymorphism, the R allele predominates in the East Asian populations (>60%), which is significantly higher than in the South Asian populations (about 40%) and the European populations (<35%). For the *PON1* L55M polymorphism, the M allele frequency is rare in the East Asian populations (<5%), which is significantly lower than in the South Asian populations (about 20%) and European populations (>30%). Such heterogeneous genetic backgrounds could be, at least in part, responsible for the heterogeneity of effect on the risk of T2DM detected in our overall population meta‐analysis. Furthermore, subgroup analysis stratified by ethnicity also indicated that the heterogeneity in the Asian group was significantly decreased.

The meta‐analysis results in the current study should be interpreted with particular caution when large between‐study heterogeneity existed. Obvious heterogeneity was present in all the genetic models for the *PON1* Q192R polymorphism in the European population subgroup. Meta‐regression was performed to evaluate the potentially important covariates exerting substantial impact on heterogeneity. Our findings have proved that the proportion of heterogeneity explained by the MAF in controls can reach as high as 93.06%. One of the reasons may be the small number of subjects in the control group. For example, the study of Elnoamany *et al*. 45 included 40 control subjects and the MAF of *PON1* Q192R was 0.213, while the study of Gokcen *et al*. 47. included 30 control subjects and the MAF of *PON1* Q192R was 0.5. Accordingly, studies with large sample size are needed to be investigated in the future.

There are some shortcomings in our current meta‐analysis. First, our included studies were limited to English and Chinese language, with some data published in other languages excluded, which may lead to some publication bias and thus affect the pooled results in the meta‐analysis. Second, although there are 34 eligible studies for the *PON1* Q192R polymorphism meta‐analysis and 16 eligible studies for the *PON1* L55M polymorphism, the populations were restricted to Asians, Europeans and Canadian Aboriginals. Studies from other populations should be conducted to confirm the findings. Last but not the least, the information about exposure to environmental substrates was not available in the included studies. This may explain some between‐study heterogeneity in our meta‐analysis. In addition, the gene×environment interactions are needed to be further evaluated in the future.

In conclusion, we have firmly established that the *PON1* genetic polymorphisms (Q192R and L55M) play important roles in the risk of T2DM with distinct effects across European and Asian populations. Further studies from other populations are needed to confirm these results.

## Conflict of interest

The authors confirm that there are no conflicts of interest.
